# Successful laparoscopic management of concomitant ectopic pregnancy and acute appendicitis in a patient of failed tubal ligation – case report with a review of the literature

**DOI:** 10.1186/1757-1626-1-412

**Published:** 2008-12-22

**Authors:** Iqbal Saleem Mir, Mir Mohsin, Anjum Malik, Basharat Ahad, Syed Suraiya Arjumand Farooq

**Affiliations:** 1 Minimal Access Surgery Unit, Government Gousia Hospital, Srinagar, Kashmir, India; 2 Government Lal Ded Hospital, Srinagar, Kashmir, India; 3 SKIMS Medical College, Srinagar, Kashmir, India

## Abstract

**Background:**

The incidence of failed abdominal bilateral tubal ligation (BTL) is quite low. Most often the pregnancy following BTL is ectopic in location. The association of concurrent acute appendicitis with ectopic pregnancy is also very rare. From 1960 till 2008 only 23 such cases of ectopic pregnancy with appendicitis managed by open surgery have been reported in the medical literature.

**Case presentation:**

We present a case in which the patient had concurrent ectopic pregnancy of the right fallopian tube with acute appendicitis after failed BTL and which was managed successfully by laparoscopic approach.

**Conclusion:**

Although the combination of ectopic pregnancy and acute appendicitis is quite rare, it is wise to rule out concurrent acute appendicitis in patients of ectopic pregnancy especially if it is on the right side due to an inter-etiological relationship. Laparoscopic management of both these pathologies can be accomplished quite successfully in properly selected cases.

## Background

India is ranked second among the most populous nations of the world. To counter the rapidly expanding population voluntary tubal sterlization of females is the most common method employed. The incidence of failure of bilateral tubal ligation (BTL) is very low and usually faulty surgical technique is the cause. Most of the times the resultant pregnancy is extra uterine in location, usually in the fimbrial end of the fallopian tube. The patient can present with features of shock or severe pain located in the pelvic area.

The coincidence of acute appendicitis with ectopic pregnancy is extremely rare. From 1960 till 2008 only 23 such cases have been reported in the medical literature [1-3]. A definite inter-etiological relationship is present especially if the ectopic pregnancy is in the right tube. The patients in these previous cases have been managed by open surgical method.

We believe the present case in which the patient had concurrent ectopic pregnancy of the right fallopian tube with acute appendicitis after failed BTL, which was managed successfully by laparoscopic approach is the first such case reported in the literature. The recent relevant literature is also reviewed in this case.

## Case presentation

A 34 year old female presented to the Government Gousia Hospital, Srinagar, Kashmir, India with pain abdomen of 36 hours duration. She was a non smoker, non alcoholic, teacher by occupation. The patient initially started with dull pain in the periumblical area. After some time the pain shifted to the right iliac fossa (RIF) and became more severe. The patient had had one bout of vomiting but continued to have nausea. There was no history of syncope. There was no other significant present history.

The patient had had her normal periods 10 days back but reported that the overall flow had been less than usual. The patient was Para 2. Both the babies were delivered by lower segment cesarean section (LSCS). The last delivery being six months back. At the time of second LSCS the patient had undergone BTL by Pomeroy's method.

On examination the patient was having mild pallor. Her pulse rate was 78 beats per minute and her blood pressure was 116/78 mm Hg. Local examination revealed rebound tenderness and guarding in the RIF. An ill defined lump was also felt in the RIF. Based on the classical history and examination and an Alvarado score of more than 7, a preliminary diagnosis of acute appendicitis was made.

All laboratory investigations were within normal limits except hemoglobin (Hb%) which was 9.6 gm%. An ultra sound examination revealed presence of free fluid in the abdomen. An extra uterine mass having features of an ectopic pregnancy was also found on the right side with empty uterine cavity. Urine sample was weakly positive for beta Human Chorionic Gonadotropin (β-HCG). Keeping the nature of emergency in view the patient was shifted to the operation theatre for immediate laparoscopy.

**Figure 1:**
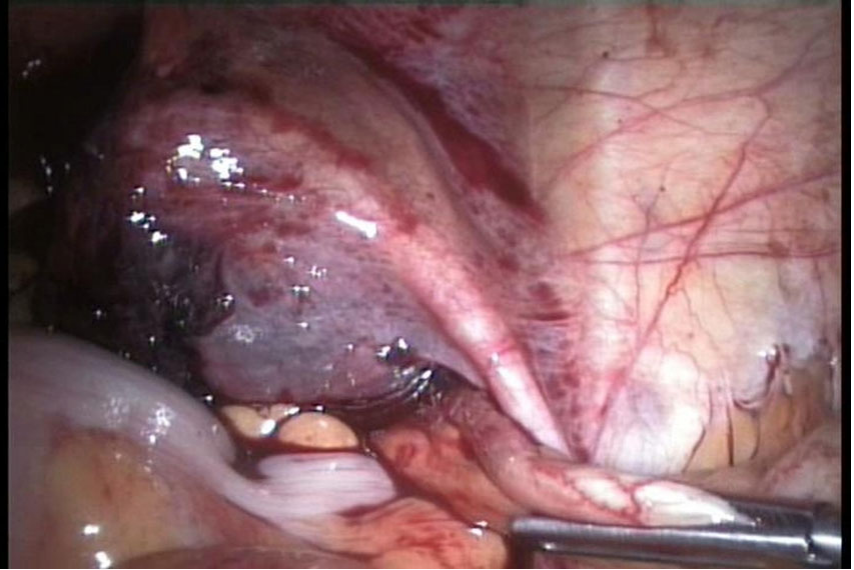
**Distended right fallopian tube with the terminal portion of the appendix engulfed in this tubo-ovarian mass**.

**Figure 2:**
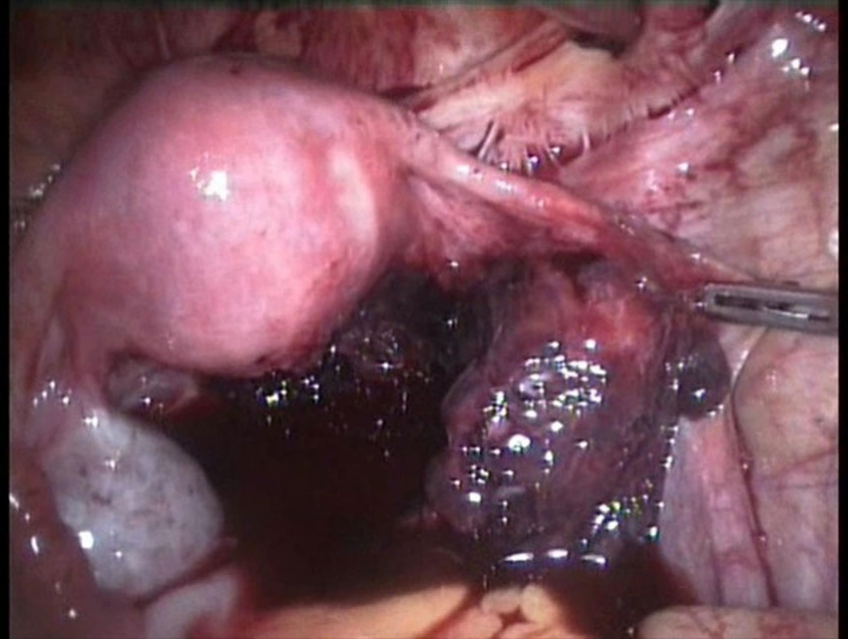
**Hemoperitoneum with right ovary adherent to the distended tube**.

**Figure 3:**
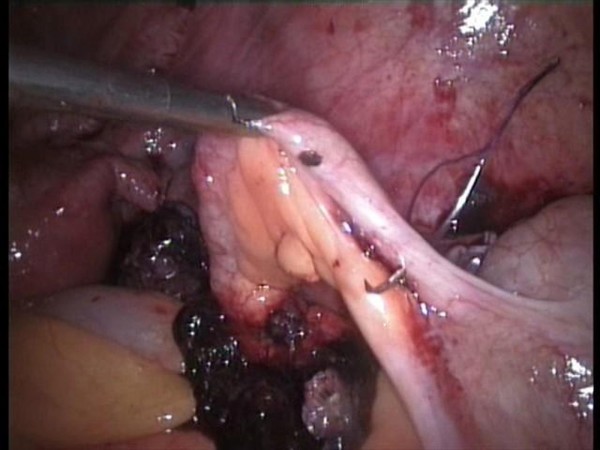
**Extensive clots adherent to the mesoappendix with features of inflammation at the tip**.

**Figure 4:**
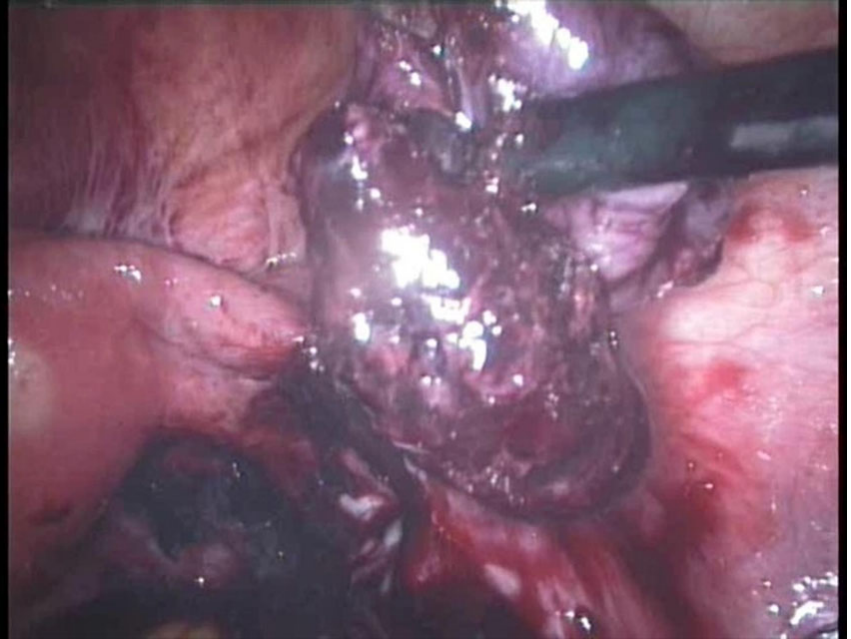
**Products of conception extruding from the tube**.

A 5 mm 30 degree telescope was used for diagnostic laparoscopy through umbilical port, which confirmed presence of hemoperitoneum. Two working ports were introduced under vision one in right hypochondrium and one in the left iliac fossa. After aspirating the blood from the pelvic area the right tube was seen to be distended. The right ovary was adherent to the tube with clots around it. The terminal portion of the appendix was engulfed in this tubo-ovarian mass (Fig. 1, 2) and was released by sharp dissection. Closer examination revealed extensive clots adherent to the mesoappendix with features of inflammation at the tip (Fig. 3). Right salpingectomy was done. During excision of the tube the products of conception extruded from the tube (Fig. 4). Laparoscopic appendicectomy was also performed using the same ports. The other tube was religated. A peritoneal lavage with 2 liters of isotonic saline was done. A tube drain was kept in place which was removed after 48 hours. The patient made an uneventful recovery.

Histopathological report of the specimen confirmed the presence of products of conception. The specimen of the appendix confirmed the presence of inflammation with extensive hemorrhage in the mesoappendix.

## Discussion

In a developing country like India which is in the late expanding phase of population cycle, tubal ligation by the open technique is the commonest method employed for achieving permanent sterilization. This procedure has a very low failure rate [4,5] and the patient can conceive more so in the first two years after ligation [6,7]. The reasons for this failure are usually faulty surgical technique, formation of fistulous tract between the severed ends or spontaneous re-anastomosis [6]. The failure rate is higher in the patients undergoing ligation at the time of cesarean section. [6]

This pregnancy is usually extra uterine or ectopic in location, most common site being the interrupted fallopian tubes [5]. It has been reported in ovaries [5] or intra abdominal locations. For the patient ectopic pregnancy after BTL can have serious consequences and even death can occur in untreated cases or where the treatment is delayed[6,8]. As the sterilization confers a sense of security the patient does not suspect pregnancy and may overlook the signs and symptoms[6]. Most often the patient has had her periods at the due date, even though the flow is scanty. This is due to the shedding of decidual cast from the uterus. This leads to a delay in seeking treatment. In cases of unruptured tubal pregnancy the patient may present with sharp agonizing pain located in the pelvic region or the iliac fossa. If tubal rupture has occurred the patient can present to the emergency room in a state of unexplained hypovolumic shock. The history of tubal ligation and the near normal monthly period can mislead even the most experienced clinician.

The laboratory investigations in case of unruptured ectopic pregnancy do not reveal any major derangement. In those patients where rupture has occurred the Hb% level and the hematocrit is low. Urinary β-HCG levels are elevated [8]. Ultrasound is the investigation of choice if patient does not have hemodynamic instability [8]. The presence of free fluid in pouch of Douglas or abdomen is significant. In such cases a tubo-ovarian mass separate from the uterus helps in arriving at a diagnosis of ectopic pregnancy especially if transvaginal USG is used [8].

The management depends upon the presentation. In patients presenting with features of shock resuscitation has to be combined with urgent laparotomy to arrest bleeding. In hemodynamically stable patients after investigations expectant treatment like use of methoteraxate or early laparoscopic management can be instituted [8,9].

Laparoscopy has both a diagnostic and a therapeutic role in the stable patient. A 5 mm telescope is ideal for diagnostic purposes to confirm hemoperitoneum or locate the ectopic pregnancy [9-12]. For successful laparoscopic management in the stable patient (hemoperitoneum < 1500 ml) additional working ports are required. The availability of laparoscopic intestinal graspers and needle holders are essential for management if the surgeon is well versed in the art of endosuturing. Otherwise harmonic shears or endoscopic linear GI staplers can be used with considerable reduction in the operation time.

### Inter-etiological relationship

The association of acute appendicitis with ectopic pregnancy is quite rare. In the internet search for literature we came across only 23 cases which have been reported till 2008. A definite inter etiological relationship has been proposed between these two abdominal pathologies. It is postulated that ectopic pregnancy may trigger appendicitis through a combination of initial inflammation and secondary infection. The authors in these case reports are of the view that ectopic pregnancy may induce contiguous inflammation, leading to periappendicitis and then appendicitis. This leads to mucosal sloughing and creates a portal for infection in the appendix by normal colonic bacterial flora[1-3]. Previously reported cases of concurrent ectopic pregnancy and appendicitis have indicated a predilection for right tubal pregnancy (75%) versus left (16%)[1,2].

The combination of ectopic pregnancy and acute appendicitis in this patient was due to the fact that the right sided tubal mass had engulfed the terminal portion of the appendix and the mesoappendix with extensive hemorrhage in it. This was the reason for the patient presenting with features of acute appendicitis.

## Conclusion

Contrary to the popular belief patients who undergo BTL can still conceive and usually this pregnancy is ectopic in location. As the patient does not seek medical help early and the history is misleading there is delay in the diagnosis and management of the patient which can lead to increased mortality and morbidity. Although the combination of ectopic pregnancy and acute appendicitis is quite rare, it is wise to rule out concurrent acute appendicitis in patients of ectopic pregnancy especially if it is on the right side due to an inter-etiological relationship. An astute surgeon should keep the diagnosis of ectopic pregnancy in mind in a patient of reproductive age group who presents with unexplained hypovolumic shock even if the patient has undergone BTL. This can reduce the mortality and morbidity associated with this surgical emergency.

Laparoscopic management of both these pathologies can be accomplished quite successfully in properly selected cases. The post operative convalescence can be reduced dramatically by laparoscopic management of these cases.

## Consent

Written informed consent was obtained from the patient for publication of this case report and accompanying images. A copy of the written consent is available for review by the Editor-in-Chief of this journal

## Competing interests

The authors declare that they have no competing interests.

## Authors' contributions

ISM, MM, AM, BA and SSAF analyzed and interpreted the patient data and all gave a major contribution in writing the manuscript. All authors read and approved the final manuscript.
